# Coenzyme Q10 and Parkinsonian Syndromes: A Systematic Review

**DOI:** 10.3390/jpm12060975

**Published:** 2022-06-15

**Authors:** Félix Javier Jiménez-Jiménez, Hortensia Alonso-Navarro, Elena García-Martín, José A. G. Agúndez

**Affiliations:** 1 Section of Neurology, Hospital Universitario del Sureste, Ronda del Sur 10, E28500 Arganda del Rey, Spain; hortalon@yahoo.es; 2 ARADyAL Instituto de Salud Carlos III, University Institute of Molecular Pathology Biomarkers, Universidad de Extremadura, E10071 Cáceres, Spain; elenag@unex.es (E.G.-M.); jagundez@unex.es (J.A.G.A.)

**Keywords:** coenzyme Q_10_, tissue concentrations, therapeutics, Parkinson’s disease, multiple system atrophy, progressive supranuclear palsy, Lewy body dementia

## Abstract

Coenzyme Q_10_ (CoQ_10_) has an important role as an antioxidant. Being that oxidative stress is one of the mechanisms involved in the pathogenesis of Parkinson’s disease (PD) and other neurodegenerative diseases, several studies addressed the concentrations of CoQ_10_ in the different tissues of patients with PD and other parkinsonian syndromes (PS), trying to elucidate their value as a marker of these diseases. Other studies addressed the potential therapeutic role of CoQ_10_ in PD and PS. We underwent a systematic review and a meta-analysis of studies measuring tissue CoQ_10_ concentrations which shows that, compared with controls, PD patients have decreased CoQ_10_ levels in the cerebellar cortex, platelets, and lymphocytes, increased total and oxidized CoQ_10_ levels in the cerebrospinal fluid and a non-significant trend toward decreased serum/plasma CoQ_10_ levels. Patients with multiple system atrophy (MSA) showed decreased CoQ_10_ levels in the cerebellar cortex, serum/plasma, cerebrospinal fluid, and skin fibroblasts. Patients with Lewy body dementia (LBD) showed decreased cerebellar cortex CoQ_10_, and those with progressive supranuclear palsy (PSP) had decreased CoQ_10_ levels in the cerebrospinal fluid. A previous meta-analysis of studies addressing the therapeutic effects of CoQ_10_ in PD showed a lack of improvement in patients with early PD. Results of the treatment with CoQ_10_ in PSP should be considered preliminary. The potential role of CoQ_10_ therapy in the MSA and selected groups of PD patients deserves future studies.

## 1. Introduction

**Figure 1:**
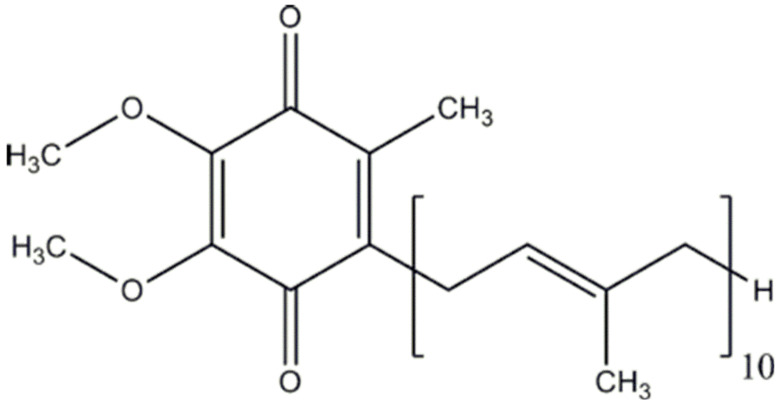
Chemical structure of coenzyme Q_10_.

Coenzyme Q_10_ (CoQ_10_, Figure 1), which is also known as ubiquinone, is a 1,4-benzoquinone that is present in the majority of tissues in the human body. It is an important component of the electron transport chain in the mitochondria, participating in the generation of cellular energy through oxidative phosphorylation. In tissues, CoQ_10_ can be present in three redox states: fully oxidized (ubiquinone), partially oxidized (semiquinone or ubisemiquinone), and fully reduced (ubiquinol). Together with mitochondria, CoQ_10_ is present in the endoplasmic reticulum, Golgi apparatus, lysosomes, and peroxisomes. CoQ_10_ has important antioxidant actions (both by scavenging free radicals and by the regeneration of other antioxidants, such as alpha-tocopherol or ascorbate acid), giving protection to cells against oxidative stress processes [1,2]. 

Because oxidative stress is one of the most important pathogenetic mechanisms of Parkinson’s disease (PD) and other neurodegenerative disorders [3,4], and because of the role of CoQ_10_ as an antioxidant, both the study of CoQ_10_ concentrations in different tissues of patients diagnosed with PD and/or other parkinsonian syndromes and the potential therapeutic role of CoQ_10_ in these diseases, have been the matter of several publications over the last two decades. The aim of this systematic review and meta-analysis is to analyze the results of studies addressing the tissular concentrations of CoQ_10_ in patients diagnosed with parkinsonian syndromes compared to healthy controls and the results of therapeutic trials of CoQ_10_ in PD and other causes of parkinsonism. 

## 2. Methods

### 2.1. Search Strategy and Criteria for Eligibility of Studies

**Figure 2:**
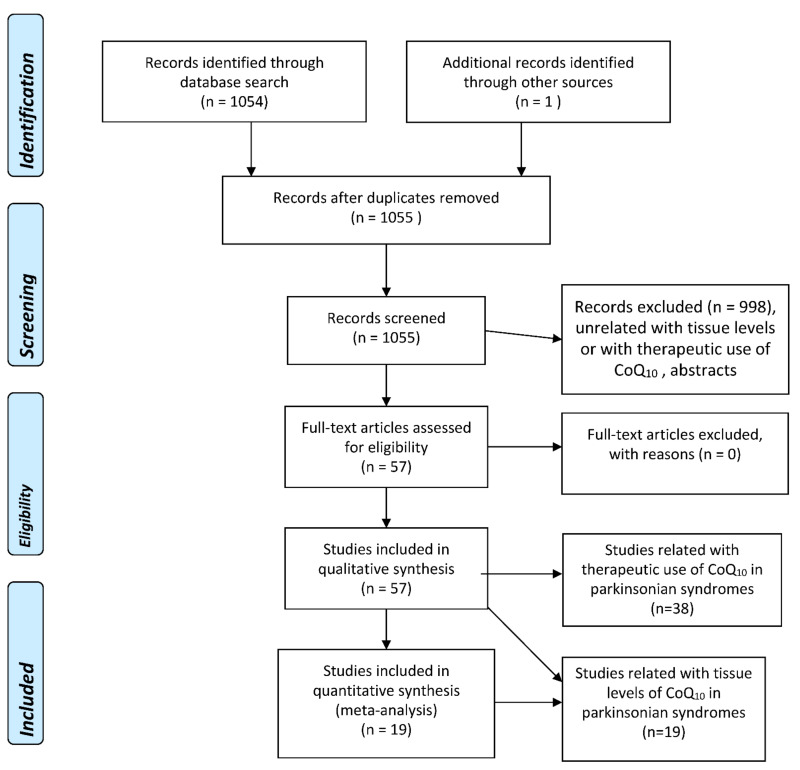
PRISMA Flowchart for the studies assessing tissue concentrations of coenzyme Q10 in parkinsonian syndromes, and for therapeutic trials with CoQ10 in parkinsonian syndromes.

A literature search using several well-known databases (PubMed, EMBASE, Web of Science (WOS) Main Collection) from 1966 until 4 May 2022, was performed. The term “coenzyme Q_10_” was crossed with “Parkinson’s disease” (356, 924, and 303 items were found in PubMed, EMBASE, and WOS, respectively), “parkinsonism” (403, 183, and 39 items were found in PubMed, EMBASE, and WOS, respectively), parkinsonian syndromes (223, 7, and 6 items were found in PubMed, EMBASE, and WOS, respectively), “multiple system atrophy” (36, 125, and 56 items were found in PubMed, EMBASE, and WOS, respectively), “Lewy body dementia” (8, 39, and 10 items were found in PubMed, EMBASE, and WOS, respectively), “Lewy body disease” (8, 62, and 24 items were found in PubMed, EMBASE, and WOS, respectively), “progressive supranuclear palsy” (31, 66, and 9 items were found in PubMed, EMBASE, and WOS, respectively), and “corticobasal degeneration” (4, 28, and 5 items were found in PubMed, EMBASE, and WOS, respectively). A total of 1054 references were retrieved by the whole search and examined one by one, and then those that were strictly related to the proposed topics, without language restrictions, were selected, excluding the duplicated articles and abstracts. The flowcharts for the selection of eligible studies—following the PRISMA guidelines [5]—analyzing tissue CoQ_10_ concentrations in patients with several types of parkinsonian syndrome and controls, and therapeutic trials with CoQ_10_ in parkinsonian syndromes, are plotted in Figure 2. 

### 2.2. Selection of Studies and Methodology for the Meta-Analyses 

Meta-analyses of those observational eligible studies that assessed the concentrations of CoQ_10_ in tissues were performed. The first author, year of publication, country, study design, and quantitative measures were extracted, and the risk of bias was analyzed by using the Newcastle–Ottawa Scale [6]. Data from selected studies analyzing the tissular concentrations of CoQ_10_ in patients diagnosed with PD compared to controls, patients diagnosed with multiple system atrophy (MSA) compared to controls, and patients with Lewy body dementia (LBD), progressive supranuclear palsy (PSP), and cortical basal degeneration (CBD) compared to healthy controls are summarized, respectively, in Table 1, Table 2 and Table 3. The plasma/serum and CSF levels of coenzyme Q10 were converted to nmol/mL, and brain tissue levels to pmol/mL, when necessary. The meta-analyses followed the PRISMA [5] (Table S1) and MOOSE guidelines [7] (Table S2) and were carried out by using the R software package meta [8]. We applied the random-effects model because of the high heterogeneity across studies, and we used the inverse variance method for the meta-analytical procedure, the DerSimonian-Laird as an estimator for Tau^2^, the Jackson method for the confidence interval of Tau^2^ and Tau, and the Hedges’ g (bias-corrected standardized mean difference). We calculated the statistical power to detect differences in mean values (alpha = 0.05) for the pooled samples when stated in the text.

## 3. Results

### 3.1. Studies Assessing Tissular CoQ_10_ Concentrations

#### 3.1.1. Parkinson’s Disease

##### Serum/Plasma

**Figure 3:**
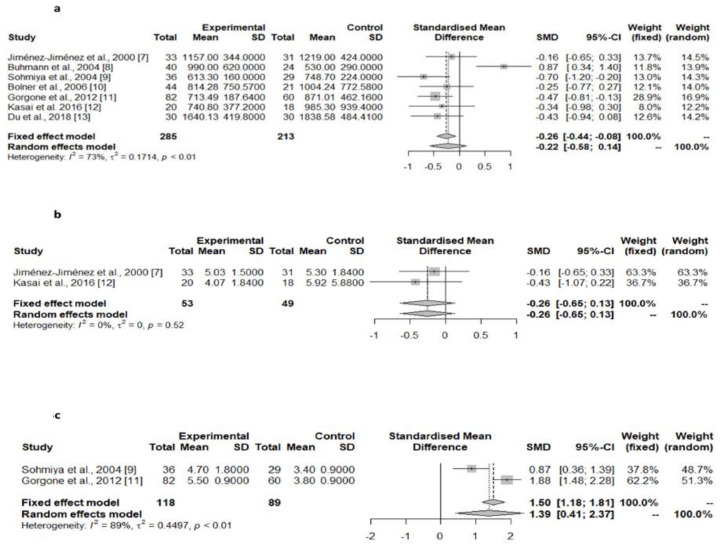
Meta-analyses of studies assessing serum/plasma total CoQ_10_ levels [7,8,9,10,11,12,13], serum/plasma CoQ_10_ corrected to cholesterol levels (**b**) [7,12], and serum/plasma oxidized CoQ_10_/total CoQ_10_ ratio (**c**) in PD patients compared with controls [9,11].

Matsubara et al. [28] reported decreased serum CoQ_10_ levels in PD patients. However, the comparison group was composed of patients with cerebral infarction instead of healthy controls. The pooled results of the seven studies assessing the serum or plasma total CoQ_10_ levels in PD patients compared with controls [9,10,11,12,13,14,15], did not show significant differences in this value between the two groups (Table 1, Figure 3a), as was the case with the two studies assessing the serum or plasma CoQ_10_ corrected to cholesterol levels (Table 1, Figure 3b) [9,14]. However, the serum/plasma oxidized CoQ_10_/total CoQ_10_ ratio was found to be significantly higher in PD patients compared with controls in two of these studies (Table 1, Figure 3c) [11,13]. Two studies showed a lack of differences in the serum/plasma oxidized CoQ_10_ and in the reduced CoQ_10_ concentrations between PD patients and controls [11,14], although there were substantial differences in these values between these studies.

##### Blood Cells

Two studies showed decreased CoQ_10_ concentrations in platelets [16] and lymphocytes [17], respectively, from patients with PD compared with healthy controls (Table 1).

##### Cerebrospinal Fluid (CSF)

According to two studies, PD patients showed increased total [18,19] and oxidized [18] CSF CoQ_10_ concentrations (Table 1).

##### Brain

**Figure 4:**
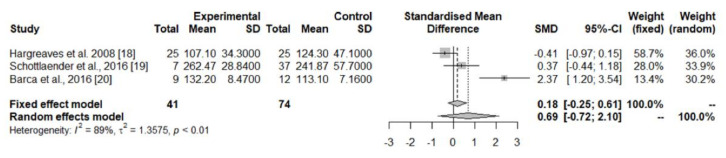
Meta-analyses of studies assessing CoQ_10_ concentrations in the cerebellar cortex of PD patients and controls [18,19,20].

The pooled data from three studies [20,21,22] showed decreased CoQ_10_ levels in the cerebellar cortex of PD patients in comparison with controls (Table 1, Figure 4), while concentrations in the cerebral cortex [20,21], striatum [20], and substantia nigra [20] did not differ significantly between the two groups (Table 1).

##### Skin Fibroblasts

Del Hoyo et al. [23] reported similar CoQ_10_ concentrations in skin fibroblasts from PD patients and controls (Table 1).

**Figure 5:**
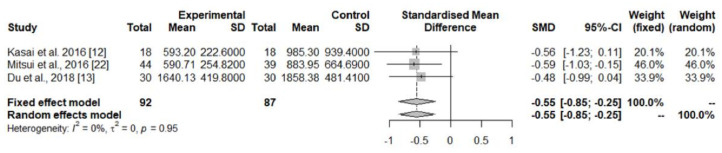
Meta-analyses of studies assessing serum/plasma total CoQ_10_ levels, serum/plasma total CoQ_10_ concentrations in MSA patients and controls [12,13,22].

The pooled data from the three studies, addressing serum/plasma total CoQ_10_ concentrations [14,15,24], showed a decrease in this value in patients with MSA compared with controls (Table 2, Figure 5). There have been reported decreased CoQ_10_ concentrations in the CSF [19], cerebellum cortex [21,22], and skin fibroblasts [25] from MSA patients (Table 2), although the pooled data from studies assessing cerebellum cortex CoQ_10_ levels did not reach statistical significance. In contrast, the cerebral cortex [21,22] and striatum [21] CoQ_10_ levels were similar in MSA and controls (Table 2).

#### 3.1.2. Other Parkinsonian Syndromes

The results of studies addressing CoQ_10_ concentrations in patients with other parkinsonian syndromes, compared with healthy controls, are summarized in Table 3. In summary, patients with *Lewy body dementia* (DLB) showed similar CoQ_10_ concentrations to those of the control in serum/plasma [26,27], and in the cerebral cortex [21], but lower CoQ_10_ concentrations in the cerebellum cortex [21]. Patients diagnosed with progressive supranuclear palsy (PSP) showed decreased CSF CoQ_10_ levels [19], and patients with cortical basal degeneration showed normal cerebral cortex CoQ_10_ levels [21].

### 3.2. Studies Assessing Therapeutic Response to CoQ_10_ Administration

#### 3.2.1. Parkinson’s Disease

The results of the 10 eligible studies addressing the therapeutic response of CoQ_10_ administration in patients with PD [29,30,31,32,33,34,35,36,37,38] are summarized in Table 4. One of these studies used an open-label design [29], while the others were randomized clinical placebo-controlled trials [30]. CoQ_10_ was generally well-tolerated, according to the four studies assessing adverse effects [32,33,34,35,36]. Despite five of these studies showing a mild improvement in motor scales in PD patients [30,31,35,36,38], three meta-analyses [39,40,41], one of them including eight randomized clinical trials [41], concluded that CoQ_10_ was not superior to the placebo in improving motor symptoms.

The study by Yoritaka et al. [38] showed a significant improvement in motor symptoms of PD patients suffering from the “wearing-off” phenomenon, and Li et al. [37] described a positive effect of concomitant CoQ_10_ and creatine therapy on cognitive impairment, assessed by the Montreal Cognitive Assessment (MoCA). However, these results are based on a small size series.

Mitsui et al. [42] reported the effects of the treatment with CoQ_10_ 1200 mg/day in a patient diagnosed with familial MSA, in an advanced stage, related to the compound heterozygous nonsense (R387X) and missense (V393A) mutations in the *COQ2* gene. The administration of CoQ_10_ resulted in increased serum and CSF total CoQ_10_ concentrations, the increased cerebral metabolic ratio of the oxygen measured by ^15^O_2_ positron emission tomography (PET), and led to stability in several clinical scores (Barthel Index, Scale for the Assessment and Rating of Ataxia—SARA, International Cooperative Ataxia Rating Scale—ICARS, and the Unified Multiple System Atrophy Rating Scale—UMSARS) during 3 years of follow-up.

#### 3.2.2. Progressive Supranuclear Palsy

Two randomized clinical trials studied the effects of CoQ_10_ in patients diagnosed with PSP. Stamelou et al. [43], in a 6-week, monocenter, double-blind, randomized, placebo-controlled, phase II trial, including 21 clinically probable PSP patients assigned to a liquid nanodispersion of CoQ10 (doses of 5 mg/kg/day) or placebo, showed a mild improvement in a Frontal Assessment Battery and in the total scores of the PSP rating scale (PSPRS) in those assigned to CoQ_10_, while there were no significant changes in the UPDRS and the Mini-Mental State Examination (MMSE). They did not describe the relevant adverse effects. As should be expected, plasma levels of CoQ10 increased in the treated, but not untreated patients. In patients receiving CoQ10 compared to those receiving the placebo, the ratio of high-energy phosphates to low-energy phosphates (adenosine-triphosphate to adenosine-diphosphate, and phosphocreatine to unphosphorylated creatine) increased significantly in the occipital lobe and showed a consistent trend towards an increase in the basal ganglia. For this reason, the authors suggested a possible disease-modifying neuroprotective of CoQ10.

In contrast, Apetauerova et al. [44], in a one-year, investigator-initiated, multicenter, randomized, placebo-controlled, double-blind clinical trial involving 61 patients diagnosed with PSP assigned to CoQ10 (2400 mg/day) or a placebo, found no significant differences between the two study groups in PSPRS (although there was a non-significant trend toward a slower decline in the CoQ_10_ group), UPDRS, activities of daily living (ADL), MMSE, the 39-item Parkinson’s Disease Questionnaire (PDQ-39), and the 36-item Short-Form Health Survey (SF-36). Despite CoQ_10_ being well-tolerated, 41% of participants withdrew from the study for different reasons.

## 4. Discussion and Conclusions

The possible role of CoQ_10_ in the pathogenesis, or its value as a diagnostic marker of PD and other parkinsonian syndromes, has not been definitively established. In the case of PD, the pooled analyses of studies measuring CoQ_10_ concentrations in the brain tissues [20,21,22], showed a significant decrease in the cerebellum cortex of PD patients (Table 1, Figure 4), which was likely related to the concentrations found in the larger control group of one of these studies [21], while CoQ_10_ concentrations in the striatum, substantia nigra, and cerebral cortex were similar in PD patients and controls. Studies in platelets [16] and lymphocytes [17] showed a consistent decrease in CoQ_10_ concentrations, while in the CSF, both the total [18,19] and oxidized CoQ_10_ levels [18] were found to be increased in PD patients. The pooled data of the seven studies assessing serum/plasma CoQ_10_ levels [9,10,11,12,13,14,15] showed a non-significant trend toward lower concentrations in PD patients compared with controls (Table 1, Figure 3a), while the percentage of oxidized vs. total CoQ_10_ was increased in PD (Table 1). One study showed a surprisingly very high percentage of oxidized CoQ_10_, which was related to the easy oxidation of the reduced to oxidized CoQ_10_ from the moment of sample extraction because precautions were not taken to prevent this oxidation [14]. Finally, CoQ_10_ levels in the skin fibroblasts were similar in PD patients and controls [23].

In MSA, CoQ_10_ concentrations were found to be decreased in the cerebellar cortex in two studies (19, 20)—although the results of the pooled data did not reach statistical significance (Table 2)—in the serum/plasma [14,15,24], CSF [19], and skin fibroblasts [25]. Patients with LBD showed decreased cerebellar CoQ_10_ [21] and patients with PSP showed decreased CoQ_10_ concentrations [19] in single studies.

Due to their antioxidant actions, it was proposed that CoQ_10_ administration could be a potential protective therapy in PD and other neurodegenerative diseases [45,46]. Moreover, the administration of CoQ_10_ has shown neuroprotective effects in several models of experimental parkinsonism:

(a) CoQ_10_ or idebenone (an analog of CoQ_10_) attenuates the loss of striatal dopamine and dopaminergic axons, induced by 1-methyl-4-phenyl-1,2,5,6-tetrahydropyridine (MPTP) administration in rodents [47,48,49,50] and in monkeys [51].
(b) In rats, both the coadministration of CoQ_10_ and creatine [52] or CoQ_10_ and nicotinamide [53] have shown additive neuroprotective effects against striatal dopamine depletion after MPTP administration.
(c) In rats injected with 6-hydroxydopamine (6-OHDA), the coadministration of CoQ_10_ and a mir-149sp mimic [54], or of CoQ_10_ and bone marrow stromal cells (BMSC) [55], improves motor symptoms and prevents dopaminergic damage.
(d) CoQ_10_ administration was also able to prevent iron-induced apoptosis in cultured human dopaminergic (SK-N-SH) neurons, in metallothionein gene-manipulated mice, and in alpha-synuclein *knockout* (*alpha-synko*) mice [56].
(e) CoQ_10_ administration can prevent neurodegeneration and behavioral deterioration in rodents exposed to several toxins causing experimental parkinsonism, such as the pesticides paraquat [57,58], dichlorvos [59], and rotenone [60,61], and showed neuroprotective effects against rotenone in primary rat mesencephalic cultures [62] and human neuroblastoma cells [63]. Interestingly, the exposure of human neuroblastoma SH-SY5Y cells to commonly used organophosphate compounds, such as dichlorvos, methyl-parathion (parathion), and chlorpyrifos (CPF), induces an important decrease in CoQ_10_ levels and complex II + III activity—both related to a decrease in neuronal cell viability. In this model, CoQ_10_ supplementation can modestly although significantly increase complex II + III activity [64].
(f) CoQ_10_ supplementation (with or without the concomitant treatment of levodopa) has shown a protective effect against chlorpromazine-induced parkinsonism in mice, including a reduction in mortality and catalepsy, an increase in dopamine levels, and a decrease in oxidative stress [65]. Similarly, CoQ_10_ improved the forced swimming test, locomotor activity test, catalepsy, muscle coordination, and akinesia test, and reduced the dopamine depletion in haloperidol-induced parkinsonism in rats [66].

However, CoQ_10_ had not shown neuroprotective effects in a *Drosophila DJ-1* model of PD [67]. Moreover, idebenone can induce apoptotic death cells in human neuroblastoma cells [68]. On the other hand, MPTP and its metabolite 1-methyl-4-phenyl-2,3-dihydropyridinium (MPDP^+^) are also able to induce a reduction in CoQ_10_, and a reduction in CoQ_10_ promotes the conversion of MPDP^+^ to the active neurotoxin 1-methyl-4-phenylpiridinium (MPP+) and increases its neurotoxicity [69].

Several studies analyzed the effects of CoQ_10_ administration on serum/plasma and CSF CoQ_10_ levels. Lönnrot et al. [70] described a significant increase in the plasma CoQ_10_ concentrations, but a lack of changes in the CSF CoQ_10_ concentrations in five healthy individuals after oral supplementation with ascorbic acid and CoQ_10._ Shults et al. [71], described an increase in plasma CoQ_10_ concentrations in 17 subjects after the administration of an escalating dosage of coenzyme Q10 (1200, 1800, 2400, and 3000 mg/day) with a stable dosage of vitamin E (alpha-tocopherol) 1200 IU/day, reaching the maximum plasma concentration with 2400 mg/day. Nukui et al. [72] reported, both in a double-blind, placebo-controlled study involving 46 healthy volunteers humans and in an acute, single-dose administration study in rats, that the administration of a water-soluble type of CoQ_10_ reached considerably higher serum CoQ_10_ concentrations than conventional CoQ_10_.

Despite the possible beneficial effects of CoQ_10_ administration, its good absorption, the lack of important adverse effects, and the improvement in PD symptoms suggested by several studies [30,31,35,36,38], data from meta-analyses of randomized clinical trials did not suggest the general usefulness of this therapy in patients with PD [39,40,41]. Several biochemical studies suggest the presence of CoQ_10_ deficiency in MSA, but the possible role of this compound in the treatment of MSA has not been explored yet. Although short-term use of CoQ_10_ treatment in PSP showed promising effects [43], the results of a randomized clinical trial involving a small series of patients showed no beneficial effects [44].

Despite all these data, the improvement in motor symptoms reported in a small series of patients with PD and the “wearing-off” phenomenon under CoQ_10_ therapy [38], and the improvement in cognitive impairment in patients treated with the combination of CoQ_10_ and creatine [37] suggest that CoQ_10_ could be useful in selected patients, and the role of personalized medicine could be important. In this regard, Seet et al. [73], in a preliminary study involving 16 PD patients treated with different doses of CoQ_10_, described that patients who experienced a significant short-term reduction in the UPDRS score had lower baseline plasma ubiquinol and decreased F2-isoprostanes (CoQ_10_ and F2-isoprostanes increased significantly at a 2400 mg/day dosage of CoQ_10_), suggesting that the therapeutic response should depend on the baseline levels of these two compounds.

Moreover, a recent double-blind randomized, phase II, placebo-controlled study using an omics-based strategy with CoQ_10_ has been recently proposed [74]. In this study, the assignation to a treatment group should be done after the stratification by the so-called “mitochondrial risk burden” in homozygous or compound heterozygous Parkin/PINK1 mutation carriers (P++), heterozygous Parkin/PINK1 mutation carriers (P+), and “omics” positive (omics+) and “omics” negative PD patients (omics-), those being omics+ with the highest and those who are omics- with the lowest cumulative burden of common genetic variants in genes that are related to mitochondrial function. Changes in the motor subscore of UPDRS should be the primary endpoint, and the appearance of motor fluctuations and non-motor symptoms in the ^31^P-magnetic resonance spectroscopy (^31^P-MRS) imaging results, and changes in structural and functional brain anatomy (MRI), should be the secondary endpoints.med-con

In summary, according to the current data, the possible value of the treatment with CoQ_10_ in parkinsonian syndromes could deserve further studies, at least in selected subgroups of patients with PD and in patients diagnosed with MSA and PSP.

## Figures and Tables

**Table 1:** Coenzyme Q10 Concentrations in Several Tissues from Parkinson’s Disease (PD) Patients and Healthy Controls (HC).

Tissue	Author, Year [Ref]	Parameter	PD N	PD Mean ± SD (Except % in *)	HC N	HC Mean ± SD	Difference in Means (95% C.I.), *p*
**Serum/plasma**	Jiménez-Jiménez et al., 2000 [9]	Total CoQ_10_ (nmol/L)	33	1157 ± 344	31	1219 ± 424	62.00 (−130.39 to 254.39); 0.522
	Buhmann et al., 2004 [10]	Total CoQ_10_ (nmol/L)	40	990 ± 620	24	530 ± 290	−460.00 (−729.67 to −190.32); 0.001
	Sohmiya et al., 2004 [11]	Total CoQ_10_ (nmol/L)	36	613.3 ± 160	29	748.7 ± 224	135.40 (40.11 to 230.69); 0.006
	Bolner et al., 2006 [12]	Total CoQ_10_ (nmol/L)	44	814.28 ± 750.57	21	1004.24 ± 772.58	189.96 (−211.60 to 591.52); 0.348
	Gorgone et al., 2012 [13]	Total CoQ_10_ (nmol/L)	82	713.49 ± 187.64	60	871.01 ± 162.16	157.52 (97.95 to 217.09); <0.001
	Kasai et al. 2016 [14]	Total CoQ_10_ (nmol/L)	20	740.8 ± 377.2	18	985.3 ± 939.4	244.50 (−217.59 to 706.59); 0.290
	Du et al., 2018 [15]	Total CoQ_10_ (nmol/L)	30	1640.13 ± 419.80	30	1838.58 ± 481.41	198.45 (−34.98 to 431.88); 0.094
	**TOTAL SERIES**	**Total CoQ_10_ (nmol/L)**	**285**	**906.01 ± 531.19**	**213**	**1025.65 ± 592.90**	**Random-effects model *p* = 0.234**
	Jiménez-Jiménez et al., 2000 [9]	Total CoQ_10_/cholesterol	33	5.03 ± 1.50	31	5.30 ± 1.84	02.7 (−0.57 to 1.11); 0.521
	Kasai et al. 2016 [14]	Total CoQ_10_/cholesterol	20	4.07 ± 1.84	18	5.92 ± 5.88	1.85 (−0.47 to 4.17); 0.115
	**TOTAL SERIES**	**Total CoQ_10_/cholesterol**	**53**	**4.67 ± 1.69**	**49**	**5.53 ± 3.80**	**Random-effects model *p* = 0.197**
	Sohmiya et al., 2004 [11]	% Oxidized/total CoQ_10_	36	4.7 ± 1.8	29	3.4 ± 0.9	−1.30 (−2.03 to −0.57); <0.001
	Gorgone et al., 2012 [13]	% Oxidized/total CoQ_10_	82	5.5 ± 0.9	60	3.8 ± 0.9	−1.70 (−2.00 to −1.40); <0.001
	**TOTAL SERIES**	**% Oxidized/total CoQ_10_**	**118**	**5.26 ± 1.29**	**89**	**3.67 ± 0.9**	**Random-effects model *p* = 0.006**
	Sohmiya et al., 2004 [11]	Oxidized CoQ_10_ (nmol/L)	36	28.3 ± 10.5	29	24.7 ± 8.3	−3.60 (−8.38 to 1.18); 0.137
	Kasai et al. 2016 [14]	Oxidized CoQ_10_ (nmol/L)	20	644.2 ± 382.4	18	900.2 ± 890.6	256.00 (−186.86 to 698.86); 0.249
	Sohmiya et al., 2004 [11]	Reduced CoQ_10_ (nmol/L)	36	585 ± 155	29	724 ± 219	139.00 (46.17 to 231.83); 0.004
	Kasai et al. 2016 [14]	Reduced CoQ_10_ (nmol/L)	20	96.6 ± 118.2	18	85.2 ± 66.6	−11.40 (−75.52 to 52.72); 0.721
**Platelets**	Götz et al., 2000 [16]	Total CoQ_10_ (ng/10^9^ platelets)	20	80.6 ± 5.9	19	93.7 ± 5.1	13.10 (9.51 to 16.69); <0.001
	Götz et al., 2000 [16]	Reduced CoQ_10_ (ng/10^9^ platelets)	20	10.3 ± 2.4	19	20.3 ± 3.2	10.00 (8.17 to 11.83); <0.001
	Götz et al., 2000 [16]	Oxidized CoQ_10_ (ng/10^9^ platelets)	20	70.3 ± 4.8	19	73.5 ± 4.7	3.20 (0.07 to 6.33); 0.045
	Götz et al., 2000 [16]	Reduced/oxidizedCoQ_10_	20	0.15 ± 0.04	19	0.32 ± 0.07	0.17 (0.13 to 0.21); <0.001
	Götz et al., 2000 [16]	Reduced/total CoQ_10_	20	0.11 ± 0.02	19	0.21 ± 0.03	0.10 (0.08 to 0.12); <0.001
**Lymphocytes**	Mischley et al., 2012 [17] *	% of patients with CoQ_10_ deficiency *	22	32–36%	88	8–9%	*p* = 0.0012–0.006 (according to authors data)
**CSF**	Isobe et al., 2007 [18]	Oxidized CoQ_10_ (nmol/L)	20	5.2 ± 1.5	17	2.9 ± 1.3	−2.30 (−3.25 to −1.35); <0.001
	Isobe et al., 2007 [18]	Reduced CoQ_10_ (nmol/L)	20	0.7 ± 0.6	17	0.8 ± 0.7	0.10 (−0.33 to 0.53); 0.643
	Isobe et al., 2007 [18]	Oxidized/total CoQ_10_	20	0.803 ± 0.179	17	0.682 ± 0.204	−0.12 (−0.25 to 0.01); 0.063
	Compta et al., 2018 [19]	Total CoQ_10_ (nmol/L)	15	54.39 ± 7.16	15	36.02 ± 7.20	−18.37 (−23.74 to −13.00); < 0.001
**Brain**							
Striatum	Hargreaves et al. 2008 [20]	Total CoQ_10_ (pmol/mg protein)	20	188.6 ± 51.4	20	214.3 ± 64.3	25.70 (−11.56 to 62.96); 0.171
Substantia nigra	Hargreaves et al. 2008 [20]	Total CoQ_10_ (pmol/mg protein)	8	102.9 ± 42.9	8	120.0 ± 4.3	17.10 (−15.59 to 49.79); 0.281
Cerebellum cortex	Hargreaves et al. 2008 [20]	Total CoQ_10_ (pmol/mg protein)	25	107.1 ± 34.3	25	124.3 ± 47.1	17.20 (−6.23 to 40.63); 0.147
	Schottlaender et al., 2016 [21]	Total CoQ_10_ (pmol/mg protein)	7	262.47 ± 28.84	37	241.87 ± 57.70	−2.06 (−65.95 to 24.75); 0.365
	Barca et al., 2016 [22]	Total CoQ_10_ (pmol/mg protein)	9	132.2 ± 8.47	12	113.1 ± 7.16	−19.10 (−26.24 to −11.96); <0.001
	**TOTAL SERIES**	Total CoQ_10_ (pmol/mg protein)	**41**	**139.14** ± 64.49	**74**	**181.27** ± 78.20	**Random-effects model** ***p* = 0.03358**
Cerebral cortex	Hargreaves et al. 2008 [20]	Total CoQ_10_ (pmol/mg protein)	13	128.6 ± 61.4	13	218.6 ± 55.7	90.00 (42.55 to 137.45); 0.0007
	Schottlaender et al., 2016 [21]	Total CoQ_10_ (pmol/mg)	7	276.02 ± 71.37	37	259.39 ± 107.09	−16.63 (−102.09 to 68.84); 0.697
	**TOTAL SERIES**	Total CoQ_10_ (pmol/mg)	**20**	**180.20** ± 99.89	**50**	**248.78** ± 97.53	**Random-effects model** ***p* = 0.143**
**Skin fibroblasts**	Del Hoyo et al., 2010 [23]	Total CoQ_10_/CS	20	1.16 ± 0.33	19	0.97 ± 0.25	−0.19 (−0.38 to 0.00); 0.051
	Del Hoyo et al., 2010 [23]	Reduced CoQ_10_/CS	20	0.41 ± 0.16	19	0.34 ± 0.11	−0.07 (−0.16 to 0.02); 0.122
	Del Hoyo et al., 2010 [23]	Oxidized CoQ_10_/CS	20	0.75 ± 0.26	19	0.63 ± 0.23	−0.12 (−0.28 to 0.04); 0.136
	Del Hoyo et al., 2010 [23]	Total CoQ_10_/mg protein	20	86.27 ± 29.07	19	71.86 ± 26.38	−14.41 (−32.45 to 3.63); 0.114
	Del Hoyo et al., 2010 [23]	Reduced CoQ_10_/mg protein	20	24.50 ± 7.38	19	24.50 ± 7.38	0.00 (−4.79 to 4.79); 1.000
	Del Hoyo et al., 2010 [23]	Oxidized CoQ_10_/mg protein	20	56.49 ± 25.20	19	47.31 ± 23.50	−9.18 (−25.01 to 6.65); 0.248
	Del Hoyo et al., 2010 [23]	Oxidized CoQ_10_/Reduced CoQ_10_	20	0.60 ± 0.27	19	0.62 ± 0.27	0.02 (−0.16 to 0.20); 0.818

* Expressed in % of patients with CoQ_10_ defficiency.

**Table 2:** Coenzyme Q10 Concentrations in Several Tissues from Patients with Multisystem Atrophy (MSA) and Healthy Controls (HC).

**Tissue**	**Author, Year [Ref]**	**Parameter**	**MSA N**	**MSA Mean ± SD**	**HC N**	**HC Mean ± SD**	**Difference in Means (95% C.I.), *p***
**Serum/plasma**	Kasai et al. 2016 [14]	Total CoQ_10_ (nmol/L)	18	593.2 ± 222.6	18	985.3 ± 939.4	392.10 (−70.34 to 854.54); 0.094
	Mitsui et al., 2016 [24]	Total CoQ_10_ (nmol/L)	44	590.71 ± 254.82	39	833.95 ± 664.69	243.24 (28.09 to 458.39); 0.027
	Du et al., 2018 [15]	Total CoQ_10_ (nmol/L)	30	1640.13 ± 419.80	30	1858.38 ± 481.41	218.25 (−15.18 to 451.68); 0.066
	**TOTAL SERIES**	Total CoQ_10_ (nmol/L)	**92**	**933.40** ± 583.47	**87**	**1218.52** ± 817.98	**Random-effects model** ***p* = 0.001**
	Kasai et al. 2016 [14]	Total CoQ_10_/cholesterol	18	3.04 ± 1.23	18	5.92 ± 5.88	2.88 (0.00 to 5.76); 0.050
	Kasai et al. 2016 [14]	Oxidized CoQ_10_ (nmol/L)	18	520.7 ± 202.8	18	900.2 ± 890.6	379.50 (−58.02 to 817.02); 0.087
	Kasai et al. 2016 [14]	Reduced CoQ_10_ (nmol/L)	18	72.4 ± 34.1	18	85.2 ± 66.6	12.80 (17.64 to 48.64); 0.473
**CSF**	Compta et al., 2018 [19]	Total CoQ_10_ (nmol/L)	20	26.63 ± 3.70	15	36.02 ± 7.10	9.37 (5.61 to 13.13); <0.0001
**Brain**							
Cerebellum cortex	Schottlaender et al., 2016 [21]	Total CoQ_10_ (pmol/mg)	20	169.30 ± 49.71	37	241.87 ± 57.70	72.57 (41.94 to 103.20); <0.001
	Barca et al., 2016 [22]	Total CoQ_10_ (pmol/mg)	12	68.1 ± 10.03	12	113.1 ± 7.16	45.00 (37.62 to 52.38); <0.001
	**TOTAL SERIES**	Total CoQ_10_ (pmol/mg)	**32**	131.35 ± 63.47	**49**	210.33 ± 75.09	**Random-effects model** ***p* = 0.0977**
Cerebral cortex frontal	Schottlaender et al., 2016 [21]	Total CoQ_10_ (pmol/mg)	20	260.44 ± 70.22	37	259.39 ± 107.09	−1.05 (−54.43 to 52.33); 0.969
Cerebral cortex occipital	Barca et al., 2016 [22]	Total CoQ_10_ (nmol/mg protein)	10	277.1 ± 29.73	9	267.3 ± 21.88	−9.80 (−35.32 to 15.72); 0.429
Striatum	Barca et al., 2016 [22]	Total CoQ_10_ (nmol/mg protein)	7	244.2 ± 27.16	7	230.8 ± 28.62	−13.40 (−45.89 to 10.09); 0.387
**Skin fibroblasts**	Monzio Compagnoni et al., 2010 [25]	Total CoQ_10_ (pg/mg protein)	14	27.83 ± 1.44	6	45.22 ± 3.48	17.39 (15.13 to 19.65); <0.001

**Table 3:** Coenzyme Q10 Concentrations in Several Tissues from Patients with Lewy Body Dementia (LBD), Progressive Supranuclear Palsy, and Cortical Basal Degeneration Compared with Healthy Controls (HC).

Lewy Body Dementia (LBD)							
Tissue	Author, Year [Ref]	Parameter	LBD N	LBD Mean ± SD	HC N	HC Mean ± SD	Difference in Means (95% C.I.), *p*
**Serum/plasma**	Molina et al., 2002 [26]	Total CoQ_10_ (nmol/L)	18	960.6 ± 359.1	20	1205.2 ± 362.2	244.60 (6.90 to 482.30); 0.044
	Gironi et al. 2011 [27]	Total CoQ_10_ (nmol/L)	7	645.17 ± 290	66	622.12 ± 227.14	−23.05 (−207.81 to 161.71); 0.804
	**TOTAL SERIES**	Total CoQ_10_ (nmol/L)	**25**	**872.28** ± 365.05	86	**757.72** ± 360.79	**Random-effects model: *p* = 0.409**
	Molina et al., 2002 [7]	Total CoQ_10_/cholesterol	18	4.67 ± 1.75	20	5.05 ± 1.52	0.38 (−0.70 to 1.46); 0.478
**Brain**							
Cerebellum cortex	Schottlaender et al., 2016 [21]	Total CoQ_10_ (pmol/mg)	20	169.30 ± 49.71	37	241.87 ± 57.70	72.57 (41.94 to 103.20); <0.001
Cerebral cortex frontal	Schottlaender et al., 2016 [21]	Total CoQ_10_ (pmol/mg)	20	260.44 ± 70.22	37	259.39 ± 107.09	−1.05 (−54.43 to 52.33); 0.969
**Progressive Supranuclear Palsy (PSP)**							
**Tissue**	**Author, Year [Ref]**	**Parameter**	**PSP N**	**PSP Mean ± SD**	**HC N**	**HC Mean ± SD**	**Difference in Means (95% C.I.), *p***
CSF	Compta et al., 2018 [19]	Total CoQ_10_ (nmol/L)	10	47.67 ± 4.05	15	36.02 ± 7.10	−11.65 (−16.79 to −6.51); 0.0001
**Cortical Basal Degeneration (CBD)**							
**TISSUE**	**Author, Year [Ref]**	**Parameter**	**CBD N**	**CBD Mean ± SD**	**HC N**	**HC Mean ± SD**	**Difference in Means (95% C.I.), *p***
Cerebellum cortex	Schottlaender et al., 2016 [21]	Total CoQ_10_ (pmol/mg)	15	271.18 ± 76.21	37	241.87 ± 57.70	−29.31 (−68.31 to 9.69); 0.137

**Table 4:** Studies describing the effects of levodopa and dopamine agonists in patients with RBD.

Authors, Year [Ref]	Study Setting	Type of Study	Main Findings	Level of Evidence (Quality Score)
Strijks et al., 1997 [29]	10 patients diagnosed with PD. Dosage of 200 mg/day. Assessment of motor performance with UPDRS and motor test.	3 months open-label study	Lack of improvement in PD motor symptoms.	II (NA)
Shults et al., 2002 [30]	Eighty subjects with early PD not requiring treatment for their disability. Dosages of 300, 600, or 1200 mg/day Evaluation with the UPDRS at the screening, baseline, and 1-, 4-, 8-, 12-, and 16-month visits. Follow-up of 16 months or until disability requiring treatment with levodopa.	Multicenter, randomized, parallel-group, placebo-controlled, double-blind, dosage-ranging trial.	Significantly lower increase in UPDRS scores during follow-up in patients assigned to CoQ_10_ therapy, especially with the highest doses.	I (>50%)
Müller et al., 2003 [31]	Twenty-eight treated and stable PD patients. Dosage of 360 mg/day for 4 weeks. Scoring of PD symptoms, and visual function using the Farnsworth–Munsell 100 Hue test (FMT).	Monocenter, parallel-group, placebo-controlled, double-blind trial	Mild symptomatic benefit on PD symptoms in patients assigned to CoQ_10_ therapy. Better improvement in FMT performance in patients assigned to CoQ_10_ therapy.	I (>50%)
NINDS NET-PD Investigators 2007 [32]	Seventy-one untreated early PD patients assigned to CoQ10 therapy (2400 mg/day), 71 to GPI-1485, and 71 to placebo. Measurement of change in total UPDRS scores and subscores, Hoehn & Yahr staging, and Schwabb & England scale scores, either at the time requiring symptomatic therapy or at 12 months.	Randomized, double-blind, calibrated futility clinical trial	The primary outcome measure (change in total UPDRS scores over 1 year) did not differ significantly between the 3 treatment groups. Changes in Hoehn & Yahr staging, and Schwabb & England scale scores did not differ significantly between the 3 treatment groups. CoQ_10_ was well-tolerated. The percentages of withdrawal because of adverse effects were 8%, 11%, and 10%, respectively, for CoQ_10_, GPI-1485, and placebo.	I (>50%)
Storch et al., 2007 [33]	One hundred thirty-one patients with PD without motor fluctuations and a stable antiparkinsonian treatment. Treatment with placebo or nanoparticular CoQ_10_ (100 mg 3 times a day, equivalent to 1200 mg/day of standard formulation) for 3 months. The stratification criterion was levodopa treatment. Evaluation with the UPDRS (sum score of parts II and III) at baseline, 1, 2, and 3 months at each visit monthly.	Multicenter, randomized, double-blind, placebo-controlled, stratified, parallel-group, single-dose trial.	The mean changes of the sum UPDRS parts II/III score did not differ significantly between the placebo and CoQ_10_ groups (−3.69 and −3.33) No secondary outcome measure showed a significant change between the placebo group and the CoQ_10_ group. The frequency and quality of adverse events are similar in both treatment groups.	I (>50%)
Parkinson Study Group QE3 Investigators [34]	Six hundred patients diagnosed with PD (from 67 hospitals in the USA) in the previous 5 years, free of dopaminergic therapy in the previous 3 months, with Hoehn & Yahr stage of 2.5 or less. Two hundred were assigned to CoQ_10_ 1200 mg/day, 200 to CoQ_10_ 2400 mg/day and 200 to placebo. All patients were taking vitamin E 1200 IU/day. Evaluation at 16 months from baseline or until a disability requiring dopaminergic treatment. The study was powered to detect a 3-point difference between active treatment and placebo.	Phase III randomized, placebo-controlled, double-blind clinical trial	At study termination, both active treatment groups showed slight adverse trends relative to placebo. Adjusted mean changes (worsening) in total UPDRS scores from baseline to final visit did not differ between the 3 study groups. Treatments were well-tolerated with no safety concerns.	I (>50%)
Jie et al., 2014 [35]	Eighty-eight patients diagnosed with PD and treated with levodopa. Forty-four were assigned to CoQ_10_ 375–750 mg/day, and 44 to placebo Evaluation with the Webster Scale at baseline and 3 months	Monocenter, randomized, placebo-controlled, double-blind clinical trial	Significant improvement in UPDRS Webster Scale scores in the group of patients treated with CoQ_10._ Lack of significant adverse effects.	I (>50%)
Wang et al., 2014 [36]	Thirty-nine patients diagnosed with PD under conventional therapy. Twenty-one were assigned to CoQ_10_ 450 or 1200 mg/day, and 18 to placebo as add-on therapy Evaluation with the UPDRS III and Webster Scale at baseline and 36 weeks	Monocenter, randomized, placebo-controlled, double-blind clinical trial	Significant improvement in UPDRS III and Webster Scale scores in the group of patients treated with CoQ_10_ 1200 mg/day (but not of the patients treated with CoQ_10_ 450 mg/day) compared with the placebo group.	I (>50%)
Li et al., 2015 [37]	Seventy-five patients diagnosed with PD and MCI. Random assignation to treatment with CoQ_10_ 100 mg b.i.d. and creatine 5 mg b.i.d. or to placebo. Evaluation with the UPDRS part III, and MoCa at 12 and 18 months.	Phase III randomized, placebo-controlled, double-blind clinical trial	Non-significant differences in UPDRS III scores between the 2 study groups at 12 and 18 months. Significantly lower worsening in the MoCA scores in patients assigned to CoQ_10_ plus creatine.	I (>50%)
Yoritaka et al., 2015 [38]	Twenty-six patients with PD experiencing wearing off (group A) and 22 early PD patients without levodopa (with or without a dopamine agonist, group B). Treatment with 300 mg/day of ubiquinol-10 or placebo for 48 weeks (Group A, 14 ubiquinol-10, 12 placeboes) or 96 weeks (Group B, 14 ubiquinol-10, 8 placeboes).	Randomized, double-blind, placebo-controlled, parallel-group pilot trial	Significant improvement in UPDRS scores in patients treated with ubiquinol-10 compared with placebo in group A. Lack of significant changes in UPDRS scores in patients treated with ubiquinol-10 compared with placebo in group B.	I (>50%)

MoCA: Montreal Cognitive Assessment, PD: Parkinson’s disease, UPDRS: Unified Parkinson’s disease rating scale.

## Data Availability

All data related to the current study, intended for reasonable use, is available from J.A.G. Agúndez (University Institute of Molecular Pathology Biomarkers, University of Extremadura -UNEx ARADyAL Instituto de Salud Carlos III, Av/de la Universidad S/N, E10071 Cáceres. Spain) and F.J. Jiménez-Jiménez (Section of Neurology, Hospital del Sureste, Arganda del Rey, Madrid, Spain).

## Supplementary Materials

The following supporting information can be downloaded at: https://www.mdpi.com/article/10.3390/jpm12060975/s1. **Table S1**. PRISMA Checklist. Table S2. MOOSE Checklist.
