# Supplementary material for: Coenzyme Q10 and Parkinsonian Syndromes: A Systematic Review
Source: J Pers Med. 2022 Jun 15;12(6):975. doi: 10.3390/jpm12060975 (PMC9225264; doi:10.3390/jpm12060975)
Supplement: Supplementary file 1 [file jpm-12-00975-s001.zip › jpm-1757585-supplementary.pdf]

COENZYME Q10 AND PARKINSONIAN SYNDROMES: A SYSTEMATIC REVIEW

Félix Javier Jiménez-Jiménez MD PhD (1), Hortensia Alonso-Navarro MD PhD (1), Elena García-Martín MD PhD (2), José A.G. Agúndez MD PhD (2).

(1) Section of Neurology, Hospital Universitario del Sureste, Arganda del Rey, Madrid. E-mails fjavier.jimenez@salud.madrid.org (F.J.J-J) and hortalon@yahoo.es (H. A-N)

(2) Universidad de Extremadura, University Institute of Molecular Pathology Biomarkers. ARADyAL Instituto de Salud Carlos III, Cáceres. SPAIN. E-mails elenag@unex.es (E.G-M) and jagundez@unex.es (J.A.G-A)

ORCID IDs:

Félix Javier Jiménez-Jiménez 0000-0002-7558-7323

Hortensia Alonso-Navarro 0000-0001-8644-2210

Elena García-Martín 0000-0002-8094-046X

José A.G. Agúndez 0000-0001-6895-9160

CORRESPONDING AUTHOR:

Félix Javier Jiménez-Jiménez

Section of Neurology, Hospital Universitario del Sureste, Ronda del Sur 10, E28500 Arganda del Rey (Madrid, SPAIN) TEL +34636968395 FAX +34913280704

E-Mails fjavier.jimenez@salud.madrid.org ; felix.jimenez@sen.es

Table S1. PRISMA Checklist.

Section/topic	#	Checklist item	Reported on page #
**TITLE**			
Title	1	Identify the report as a systematic review, meta-analysis, or both.	1
**ABSTRACT**			
Structured summary	2	Provide a structured summary including, as applicable: background; objectives; data sources; study eligibility criteria, participants, and interventions; study appraisal and synthesis methods; results; limitations; conclusions and implications of key findings; systematic review registration number.	NA
**INTRODUCTION**			
Rationale	3	Describe the rationale for the review in the context of what is already known.	3
Objectives	4	Provide an explicit statement of questions being addressed with reference to participants, interventions, comparisons, outcomes, and study design (PICOS).	3
**METHODS**			
Protocol and registration	5	Indicate if a review protocol exists, if and where it can be accessed (e.g., Web address), and, if available, provide registration information including registration number.	NA
Eligibility criteria	6	Specify study characteristics (e.g., PICOS, length of follow-up) and report characteristics (e.g., years considered, language, publication status) used as criteria for eligibility, giving rationale.	3,4
Information sources	7	Describe all information sources (e.g., databases with dates of coverage, contact with study authors to identify additional studies) in the search and date last searched.	3,4
Search	8	Present full electronic search strategy for at least one database, including any limits used, such that it could be repeated.	3,4
Study selection	9	State the process for selecting studies (i.e., screening, eligibility, included in systematic review, and, if applicable, included in the meta-analysis).	3,4
Data collection process	10	Describe method of data extraction from reports (e.g., piloted forms, independently, in duplicate) and any processes for obtaining and confirming data from investigators.	3,4
Data ítems	11	List and define all variables for which data were sought (e.g., PICOS, funding sources) and any assumptions and simplifications made.	3,4
Risk of bias in individual studies	12	Describe methods used for assessing risk of bias of individual studies (including specification of whether this was done at the study or outcome level), and how this information is to be used in any data synthesis.	3,4
Summary measures	13	State the principal summary measures (e.g., risk ratio, difference in means).	4
Synthesis of results	14	Describe the methods of handling data and combining results of studies, if done, including measures of consistency (e.g., I^2) for each meta-analysis.	4

Section/Topic	#	Checklist item	Reported on page #
Risk of bias across studies	15	Specify any assessment of risk of bias that may affect the cumulative evidence (e.g., publication bias, selective reporting within studies).	3,4
Additional analices	16	Describe methods of additional analyses (e.g., sensitivity or subgroup analyses, meta-regression), if done, indicating which were pre- specified.	NA
**RESULTS**			
Study selection	17	Give numbers of studies screened, assessed for eligibility, and included in the review, with reasons for exclusions at each stage, ideally with a flow diagram.	3-5, Tables 1-4, Figures 3-5
Study characteristics	18	For each study, present characteristics for which data were extracted (e.g., study size, PICOS, follow-up period) and provide the citations.	3-5, Tables 1-4, Figures 3-5
Risk of bias within studies	19	Present data on risk of bias of each study and, if available, any outcome level assessment (see item 12).	3-5, Tables 1-4, Figures 3-5
Results of individual studies	20	For all outcomes considered (benefits or harms), present, for each study: (a) simple summary data for each intervention group (b) effect estimates and confidence intervals, ideally with a forest plot.	3-5, Tables 1-4, Figures 3-5
Synthesis of results	21	Present results of each meta-analysis done, including confidence intervals and measures of consistency.	3-5, Tables 1-4, Figures 3-5
Risk of bias across studies	22	Present results of any assessment of risk of bias across studies (see Item 15).	3-5, Tables 1-4, Figures 3-5
Additional análisis	23	Give results of additional analyses, if done (e.g., sensitivity or subgroup analyses, meta-regression [see Item 16]).	NA
**DISCUSSION**			
Summary of evidence	24	Summarize the main findings including the strength of evidence for each main outcome; consider their relevance to key groups (e.g., healthcare providers, users, and policy makers).	8-11
Limitations	25	Discuss limitations at study and outcome level (e.g., risk of bias), and at review-level (e.g., incomplete retrieval of identified research, reporting bias).	8-11
Conclusions	26	Provide a general interpretation of the results in the context of other evidence, and implications for future research.	10, 11
**FUNDING**			
Funding	27	Describe sources of funding for the systematic review and other support (e.g., supply of data); role of funders for the systematic review.	11

From: Moher D, Liberati A, Tetzlaff J, Altman DG, The PRISMA Group (2009). Preferred Reporting Items for Systematic Reviews and Meta-Analyses: The PRISMA Statement. PLoS Med 6(7): e1000097. doi:10.1371/journal.pmed1000097
For more information, visit: www.prisma-statement.org.

Table S2. MOOSE Checklist

	Topic	Page number
Title	Identify the study as a meta-analysis (or systematic review)	1
Abstract	Use the journal's structured format	NA
Introduction	**Present:**	
	The clinical problem	3-4
	The hipótesis	3-4
	A statement of objectives that includes the study population, the condition of interest, the exposure or intervention, and the outcome(s) considered	3-4
Sources	**Describe:**	
	Qualifications of searchers (eg, librarians and investigators)	3-4
	Search strategy, including time period included in the synthesis and keywords	3-4
	Effort to include all available studies, including contact with authors	3-4
	Databases and registries searched	3-4
	Search software used, name and version, including special features used (e.g. explosion)	3-4
	Use of hand searching (e.g, reference lists of obtained articles)	3-4
	List of citations located and those excluded, including justification	3-4
	Method of addressing articles published in languages other than English	3-4
	Method of handling abstracts and unpublished studies	3-4
	Description of any contact with authors	NA
Study Selection	**Describe**	
	Types of study designs considered	3-4
	Relevance or appropriateness of studies gathered for assessing the hypothesis to be tested	3-4
	Rationale for the selection and coding of data (eg, sound clinical principles or convenience)	3-4
	Documentation of how data were classified and coded (eg, multiple raters, blinding, and inter-rater reliability)	3-4
	Assessment of confounding (e.g. comparability of cases and controls in studies where appropriate)	3-4
	Assessment of study quality, including blinding of quality assessors; stratification or regression on possible	3-4
	Assessment of heterogeneity	3-4
	Statistical methods (eg, complete description of fixed or random effects models, justification of whether the chosen models account for predictors of study results, dose-response models, or cumulative meta-analysis) in sufficient detail to be replicated	3-4

Results	**Present**	
	A graph summarizing individual study estimates and the overall estimate	Figures 3-5
	A table giving descriptive information for each included study	Tables 1-4
	Results of sensitivity testing (eg, subgroup analysis)	3-5, Tables 1-4, Figures 3-5
	Indication of statistical uncertainty of findings	3-4
Discusión	**Discuss**	
	Strengths and weaknesses	8-11
	Potential biases in the review process (eg, publication bias)	8-11
	Assessment of quality of included studies	8-11
	Consideration of alternative explanations for observed results	8-11
	Generalization of the conclusions (ie, appropriate for the data presented and within the domain of the literature review)	8-11
	Guidelines for future research	8-11
	Disclosure of funding source	11

*Modified from Stroup DF, Berlin JA, Morton SC, Olkin I, Williamson GD, Rennie D, et al. Meta-analysis of observational studies in epidemiology: a proposal for reporting. Meta-analysis Of Observational Studies in Epidemiology (MOOSE) group. JAMA 2000;283:2008–12. Copyrighted © 2000, American Medical Association. All rights reserved.
